# Candidate loci involved in domestication and improvement detected by a published 90K wheat SNP array

**DOI:** 10.1038/srep44530

**Published:** 2017-03-22

**Authors:** Lifeng Gao, Guangyao Zhao, Dawei Huang, Jizeng Jia

**Affiliations:** 1 Key Laboratory of Crop Gene Resources and Germplasm Enhancement, MOA, the National Key Facility for Crop Gene Resources and Genetic Improvement, Institute of Crop Science, CAAS, Beijing, 100081, China; 2 Beijing Institute of Genomics, Chinese Academy of Sciences, Beijing, 100101, China

## Abstract

Selection is one of the most important forces in crop evolution. Common wheat is a major world food crop and a typical allopolyploid with a huge and complex genome. We applied four approaches to detect loci selected in wheat during domestication and improvement. A total of 7,984 candidate loci were detected, accounting for 23.3% of all 34,317 SNPs analysed, a much higher proportion than estimated in previous reports. We constructed a first generation wheat selection map which revealed the following new insights on genome-wide selection: (1) diversifying selection acted by increasing, decreasing or not affecting gene frequencies; (2) the number of loci under selection during domestication was much higher than that during improvement; (3) the contribution to wheat improvement by the D sub-genome was relatively small due to the bottleneck of hexaploidisation and diversity can be expanded by using synthetic wheat and introgression lines; and (4) clustered selection regions occur throughout the wheat genome, including the centromere regions. This study will not only help future wheat breeding and evolutionary studies, but will also accelerate study of other crops, especially polyploids.

Selection, one of the most important drivers of variation in crop domestication and improvement, usually leaves genomic footprints known as selection signatures^1^. The effort, experience, intelligence and wisdom of farmers and thousands of breeders worldwide during crop domestication and improvement are hidden in the signatures. Therefore, identification of selection signatures and construction of a genome-wide selection map will produce a “treasure map” for breeders. Detection of selection signatures is a central challenge for both evolutionary biology and crop breeding.

Domestication and improvement (post-domestication selection) are important processes of crop evolution whereby selection is the major driver of adaptation to diverse environments in the achievement of high yields. “Domestication syndrome” characters, including loss of seed dispersal mechanisms, increased grain size, loss of sensitivity to environmental cues for germination and flowering, synchronous ripening, and a compact growth habit are adaptive traits selected by mankind^2^. Improvement, or plant breeding, as another evolutionary force, creates superior plant genotypes that are selected by phenotype and become fixed in cultivars with improved yield, stability, nutritional qualities, and other traits of commercial value^3^. Selection operates at specific loci and leaves its signature in distinct chromosomal regions. By comparing genomic patterns and levels of variability across populations it is possible to identify selection signatures left by evolutionary forces^4^,^5^.

Genome-wide surveys are important means of detecting deviations from neutrality among loci. With recent rapid developments in technology many plant species have been sequenced, and even re-sequenced, and millions of single nucleotide polymorphisms (SNPs) were identified. SNPs have been used to screen for selected regions across the whole genomes of rice^6^,^7^, maize^8^,^9^, soybean^10^,^11^,^12^, and tomato^13^. Most previous studies reported reduced polymorphism in relation to domestication and improvement. A well-known example is Tanksley’s funnel type selection model^14^. By using this approach it was estimated that selected loci accounted for 5% of the maize genome^15^,^16^ and 7% of the sunflower genome^17^.

In addition to diversity loss^18^,^19^, looking for extreme patterns of population differentiation (*F*_ST_)^20^,^21^ and significant changes in allele frequency and phenotype^22^ were also employed to detect loci subjected to selection. However, all these methods were used separately, and the results were not easily compared. Importantly, few selection studies in polyploid crops have been reported although more than 70% of crops species are polyploids^23^. This means that the contribution and potential of sub-genomes in polyploid crops to domestication and improvement have not been revealed.

Common wheat, a typical polyploid and a major world food source for 40% of the world population, is extensively grown on 17% of the world cropping area from 67°N in Norway, Finland and Russia to 45°S in Argentina^24^. With the millions of SNPs now discovered by next generation sequencing technology, it is possible to screen for genomic regions in wheat that have undergone selection during evolution. Currently, several wheat SNP arrays are publically available^25^,^26^ and some selected regions, or QTLs, have been identified using these SNP chips^25^,^27^.

In the present work, genome-wide surveys for molecular signatures of selection were carried on 31,417 informative SNPs by four methods. We used three wheat populations representative of two evolutionary stages, from wild relatives to landraces through domestication and to modern varieties through post-domestication selection or breeding. Loci with decreased, increased, and even constant variation were considered. A first generation map of selection signatures of wheat was constructed. Utilization of genomic regions that had undergone selection during wheat domestication and improvement is discussed.

## Results

### Detection of selection candidate loci

As previously described^25^,^28^, genotyping of wheat by the 90K Infinium wheat chip is complicated by the homoeologous genomes. Of 81,587 SNPs analysed, 34,317 were classified as AA or BB alleles after removing those with greater than 20% missing data points. The numbers of polymorphic SNPs in populations of wild accessions (*W*), landrace accessions (*L*) and modern (*M*) varieties were 30,577, 21,831 and 24,029, respectively. A subset of 82 modern varieties was randomly taken from the 429 modern varieties and 24,588 polymorphic SNPs were observed in this subset. The result ascertained that the higher number of polymorphic SNPs in modern cultivars was not due to the difference in sample size. This tendency was also observed previously where a 9K SNP array was applied to assess the diversity of landraces and modern cultivars^25^. Another explanation for the higher polymorphic SNP number in modern Chinese varieties than in landraces was the contribution of introduced varieties used in wheat breeding.

The numbers of polymorphic SNPs detected in pairwise comparisons of *WL* and *LM* were 33,403 and 26,519, respectively. Genetic differentiation between groups *W* and *L* revealed by these SNPs was about two-fold higher (*F*_ST_ = 0.148 ± 0.006) than between groups *L* and *M (F*_ST_ = 0.076 ± 0.004). Of the 34,317 polymorphic SNPs, 22,533 had known positions on the 21 chromosomes (Table 1). The number of SNPs analysed in the D sub-genome (3,800) was about one-third of those in the A and B sub-genomes (8,308 and 10,425, respectively), presumably the result of the population bottleneck effect following hexaploidisation. All 34,317 SNPs were used for detection of selection outliers between populations by using the following four approaches: F-statistical test (*F*_ST_), diversity test (*lnRH*), frequency-based test (*Freq*) and genome-wide association study (GWAS) test.

#### Selection of outlier loci by F-statistical test

**Figure 1:**
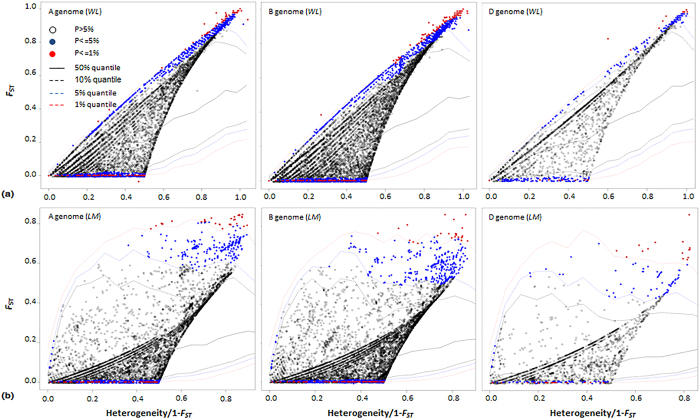
Outliers detected by the *F*_ST_ method in pairwise comparisons of *WL* (**a**) and *LM* (**b**). For SNP names of putative outliers potentially affected by selection, see Supporting Information (Supplementary Dataset 1). Y-axis, *F*_*ST*_measures locus-specific genetic divergence between populations; X-axis, Heterogeneity/1-*F*_*ST*_is a modified measure of heterogeneity per locus.

To identify loci that made significant contributions to population divergence, we used the Fdist test method available in Arlequin v3.5 to detect selection outliers. Only loci with extremely high *F*_ST_ and heterogeneity values were considered to be positive signals affecting population divergence. Respectively, 2,788 and 997 SNPs were identified as outliers in pairwise *WL* and *LM* comparisons at 5% quantile values (Fig. 1, Supplementary Dataset 1) accounting for about 8 and 3% of all 34,317 polymorphic SNPs.

#### Selection of outlier loci by diversity test

**Figure 2:**
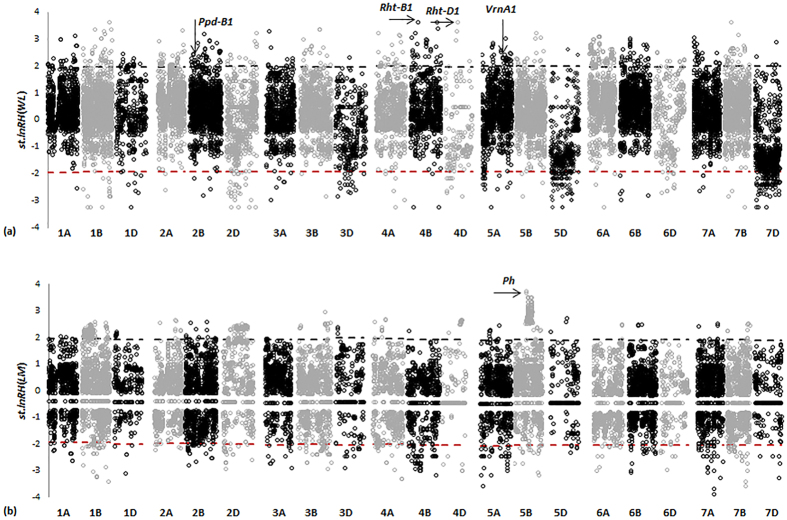
Selection candidates identified by the *lnRH* method for domestication (a) and improvement (b). Standardized *lnRH* values at the level of P < 0.05 are shown as black and red dashed lines, respectively. Loci with *st.lnRH* values beyond the region [−1.96, +1.96] are considered as selection candidates. Genetic positions are shown for *Ppd-B1, Rht-B1, Rht-D1* and *Vrn-A1* where selection candidates were identified during wheat domestication. A genetic region on chromosome 5B associated with plant height (Ph) is indicated.

Genetic variation during evolution is an important parameter of selection signatures often employed in detection of selection loci. We calculated *lnRH* values in pairwise comparisons of populations and determined confidence intervals of the *lnRH* distribution (Table 1, Supplementary Dataset 2). This method was previously confirmed as being efficient in identifying selection signatures based on SNP markers^29^. Of the 34,317 SNPs, 1,680 and 1,708 loci were detected as selection signatures during domestication and improvement, respectively (Fig. 2). Genetic variation was decreased at 1,685 loci and increased at 1,703 loci during wheat evolution.

#### Selection of outliers by the frequency-based method

**Figure 3:**
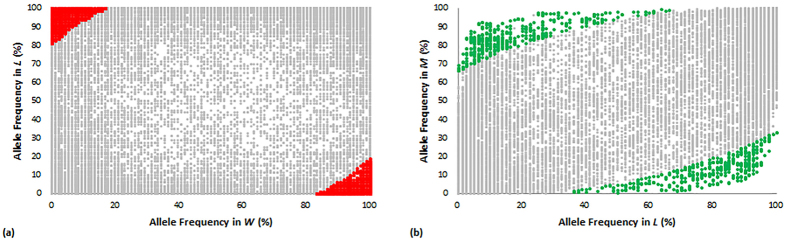
Distribution of allelic frequencies between groups. Comparisons between groups *W* and *L* (**a**) and between groups *L* and *M* (**b**). Selection candidates by domestication and improvement are shown in red and green, respectively.

Traditional selection is mainly based on phenotypes conferred by allelic variation. Therefore, variation in allele frequency in different populations can be used to detect selected loci. In this study, the *μ* test was applied to compare differences in allelic frequency between populations. Strict criteria with *μ* values higher than 11 were used to compare different loci in order to reduce the number of false positives; 1,739 and 1,035 SNPs were identified as selection candidates during domestication and improvement, respectively (Table 1 and Supplementary Dataset 3). As shown in Fig. 3, the candidates identified by pairwise comparisons demonstrated extremely different allelic frequencies between groups.

#### Phenotypic variation detected by association analysis

**Figure 4:**
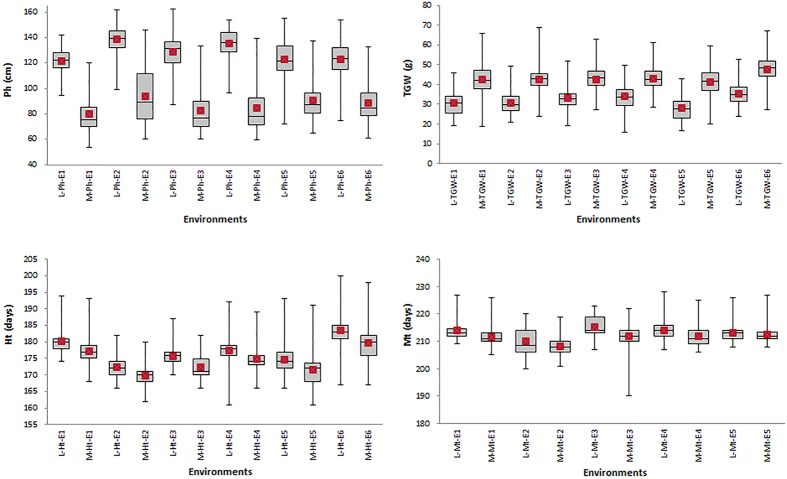
Boxplots for agronomic traits measured in different environments. Environments E1 to E4, Beijing, Jiaozuo, Luoyang and Xinxiang in crop season 2011–2012; E5 and E6, Xinxiang in 2012–2013 and 2013–2014, respectively.

During domestication and improvement selection was based on agronomically important traits, such as seed dormancy, heading time, resistance to lodging, and yield and its components. Therefore, phenotypic differences should exist between populations. Detecting such phenotypic variation and identifying the underlying genes is a direct way of finding selection signatures. This was suggested as a bottom-up approach to identify genes under selection^22^. As common wheat is hexaploid and its ancestors are diploids their phenotypes are not comparable. Therefore, phenotypic variation was assessed only between the *L* and *M* groups. Ten agronomic traits were analysed and all showed significant differences in at least three of the six environments in which the materials were grown (Table 2, Fig. 4). As expected, the plant heights (Ph) of accessions in population *M* were less than those in *L*, and likewise heading and maturity times (Ht and Mt) were also different. Moreover, the mean values for thousand grain weight (TGW) and yield (Y) of population *M* were higher than those for *L*. Population *M* was more resistant to powdery mildew (PM) than *L*. These distinct differences reflected the selective pressures of modern breeding. GWAS was conducted to find loci affecting trait differences between populations. The Q-Matrix generated from STRUCTURE was used to correct GWAS (Supplementary Fig. 1). A total of 2,507 SNPs was associated with traits using A–D test at the Bonferroni-corrected threshold (−log(P-value) ≥ 5.84, α = 0.05) (Supplementary Dataset 4, Supplementary Figs 2 to 11). Of the 2,507 selection candidates identified by GWAS, 893 were also detected by one or more of the other three methods (419, 559 and 304 loci detected by the *F*_ST_, *lnRH* and *Freq* method, respectively), accounting for 35.6% of the improvement loci (Table 3). Because only ten agronomic traits were analysed, more overlapping loci would be identified if more agronomic traits and the *W* population were assayed.

**Figure 5:**
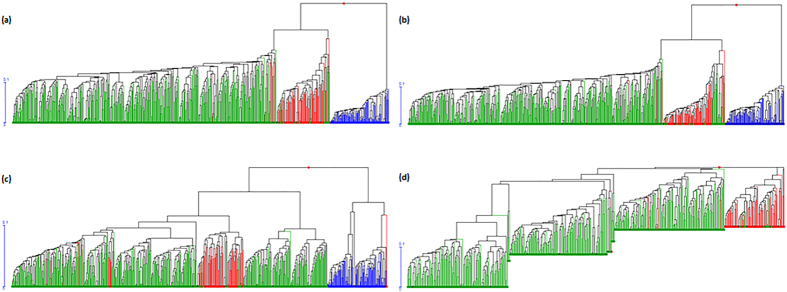
UPGMA trees of 96 wild accession combinations (*W*), 84 landraces (*L*) and 429 modern cultivars (*M*). Trees were constructed based on selection loci detected by *F*_ST_ (**a**), *Freq* (**b**), *lnRH* (**c**) and GWAS (**d**). Blue, red and green dots represent wild relatives (*W*), landraces (*L*) and modern cultivars (*M*), respectively.

Since the germplasm was divided into *W, L* and *M* groups the results should reflect the effects of artificial selection and recent breeding. To test if the loci detected by the four approaches were selected loci, we made UPGMA trees of 96 wild accessions (*W*), 84 landraces (*L*) and 429 modern cultivars (*M*) using the above candidate SNP markers. The UPGMA trees were consistent with expectation; screening by the four methods clearly separated the populations (Fig. 5), indicating the respective roles of selection in population differentiation. The distance between *W* and *L* was much larger than that between *L* and *M* because the evaluation time between *W* and *L* was much longer (8000 vs. 100 years^30^). Higher genetic differentiation was confirmed in the transition from wild species to landraces than that from landraces to modern cultivars (*F*_ST_, 0.15 *vs.* 0.08). These results proved that all of the four methods were effective in detecting selection loci.

In summary, four approaches to estimate changes in diversity, allele frequency, population divergence and GWAS, were employed to detect selection loci. In total, 7,984 candidate selection loci were detected by the four approaches, accounting for 23.3% of all 34,317 SNPs analysed, much higher than estimates in previous reports. Table 3 summarizes the number of selection candidates detected by different methods. Except for *F*_ST_ and *Freq*, common loci detected by any two approaches were less than 50%, indicating relative independence of the different methods (Table 3). This suggests that the number of selection loci detected by any individual method is incomplete. Tajima’s D tests confirmed these results. Tajima’s D is 0 for neutral variation, positive for balancing selection, and negative for selective sweep^31^. Of the 6,224 selection candidates, Tajima’s D was positive and negative for 5,817 and 407 SNPs, respectively (Supplementary Dataset 5). Of the 5,817 candidates with positive Tajima’s D, 4,125 SNPs showed significant deviations from neutrality (Tajima’s D > 1.7, p < 0.1). These 4,125 candidates were identified either by the diversity-based or frequency-based methods, or both.

### Construction of a selection map and analysis of selection candidates

**Figure 6:**
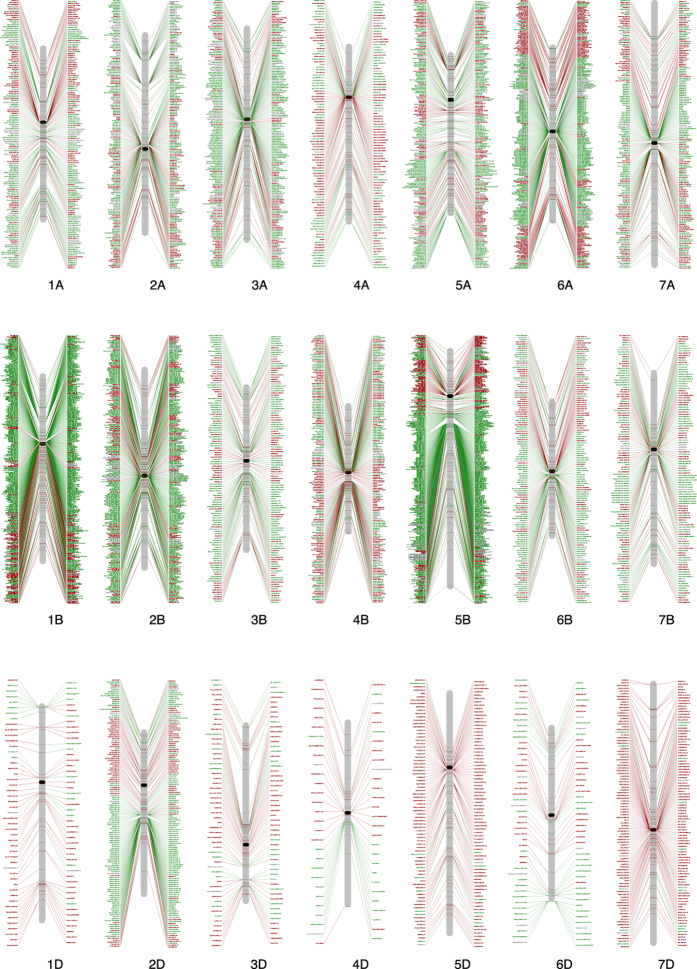
Selection map of wheat during domestication and improvement. Red, green and purple lines denote candidates subjected to selection during domestication, improvement and both processes, respectively. Centromeric regions are indicated by black dots in accordance with Cavanagh *et al*.^25^. Detailed information on the mapped loci is provided in Supplementary Datasets 1 to 4.

Construction of a selection map is of significance for both evolutionary studies and breeding. Detection of selection loci genome-wide provides an opportunity to construct a map of selection loci. Of the 7,984 candidates, 6,224 SNPs had known chromosome positions (Supplementary Datasets 1, 2, 3, 4), based on which a wheat primary selection map was constructed (Fig. 6). It is a first selection map in a crop species. This map revealed new insights into the genome-wide selection loci during wheat domestication and improvement.

### Genetic variation among selection candidates

Of the 7,984 selection locus outliers, 3,344 (42.8%) were identified by comparing diversity changes between populations with 1,659 increased and 1,641 decreased, respectively. A further 4,640 loci were identified by at least one of the other three methods. Nearly 60% of these candidates showed no statistical difference in diversity in pairwise comparisons. Results based on diversity changes suggest that selection could increase diversity and that loci with reduced variation represented only a part of the selection outliers.

The results clearly indicated that selection resulted in reduced diversity because of population bottlenecks, or hitchhiking effects (linkage drag), or the commonly known directional selection. In addition, adaptive selection also enables plants to adapt to new habitats. For example, SNP wsnp_Ex_rep_C66689_65011117 positioned in the *Vrn-A1* region on chromosome 5A was identified as a domestication candidate. This locus was monomorphic in wild relatives but showed increased diversity in modern cultivars (*He*, 0.4). More often, the diversity index did not change significantly, although allele frequencies underwent large changes.

To trace the variation in diversity among selection candidates during evolution, we found that 752 loci selected by domestication were fixed during wheat improvement. On the other hand 304 loci detected as selection targets by improvement were monomorphic during domestication, suggesting that diversity increases occurred during wheat improvement (Supplementary Dataset 2). This implied that modern breeding was a process of fixing and mining key genomic selection regions.

### The number of domestication loci is greater than the number of improvement loci

In total, 4,011 domestication loci and 2,697 improvement loci were identified at P < 0.05 by all methods except GWAS, which was used for detecting differences in the *L* and *M* populations. The total number of selected domestication loci was about 1.5-fold that of improvement loci. The numbers of loci selected during domestication and detected by *F*_ST_ and *Freq* tests was about 2.8- and 1.7-fold of that during improvement (Table 1). This was consistent with wheat evolutionary history whereby domestication lasted for more than 8,000 years whereas improvement has been for only about 100 years^30^. This explains why the distance between *W* and *L* is much larger than that between *L* and *M* in the phylogeny tree (Fig. 5). We identified 566 SNPs responsible for the separation of all three populations (Supplementary Dataset 6), suggesting continuous selection on these loci during the two periods. Clearly, selection in both processes focused on yield, adaptation and other agronomic traits. For example, we found 33 loci significantly associated with plant height (Ph), TGW, yield (Y) and heading time (Ht) in the following analyses.

### Fewer loci selected by improvement were discovered in the D sub-genome

Comparing the distribution of selection candidates among sub-genomes, 13.1, 10.2, and 17.7% of loci in the A, B, and D sub-genomes, respectively, were detected as candidates selected by domestication. In relative terms the B sub-genome contributed only in a small way compared to the D sub-genome during domestication. However, only 6.8% of loci in the D sub-genome were detected to be selection candidates during improvement compared to 7.7 and 11.0% of loci in the A and B sub-genomes, respectively (Table 1). Of the total 2,050 improvement loci, candidate loci contributed by the D sub-genome accounted for 12.5%, much fewer than that by the A (31.3%) and B (56.1%) sub-genomes, indicating a comparatively low contribution of the D sub-genome to wheat improvement. The major reason was lower polymorphism (60%) between the *L* and *M* populations in the D sub-genome than in the A (96%) and B (97%) sub-genomes, respectively, hence confirming the D sub-genome bottleneck effect in wheat improvement.

Since genetic diversity is very rich in *Aegiolops tauschii,* the donor of the D sub-genome to hexaploid wheat, there should have favorable, but unexploited, agronomically important alleles. To confirm that speculation we analysed the QTLs in introgression line derivatives of synthetic line Am3, a synthetic wheat, backcrossed to Laizhou 953, a Chinese commercial variety. Forty-eight QTL conferring nine agronomic traits were detected in three environments. Seventeen, 17 and 14 were mapped to the A, B, and D sub-genomes, respectively. The number of QTL mapped to the D sub-genome was similar to those for the A and B sub-genomes, suggesting that the D sub-genome could make a similar contribution to wheat improvement, and that fewer agronomic trait loci detected in the D sub-genome in GWAS analysis was due to the bottleneck effect of the D sub-genome during wheat domestication. Among the QTLs mapped on the D sub-genome there were favorable alleles for agronomic traits, including increased yield and TGW, reduced plant height, and resistance to powdery mildew. For example, a QTL from Am3 on chromosome 6D increased yield by 16% in three environments. This allele was not detected in the GWAS analysis of the *L* and *M* populations, suggesting that it is novel and therefore potentially valuable for future wheat improvement.

### Selection loci are distributed in clusters across the wheat genome

**Figure 7:**
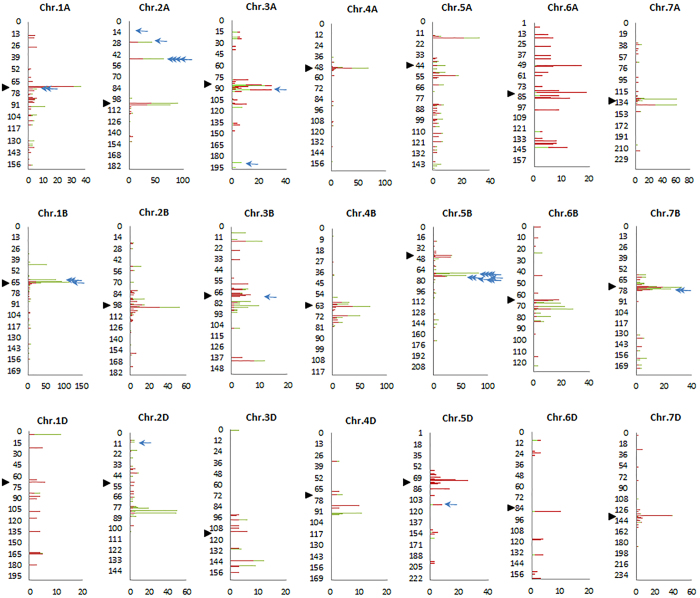
Selection candidates clustered along chromosomes. Only chromosome positions where at least three selection loci were identified are shown. Red and green bars are domestication and improvement loci, respectively. Blue arrows indicate chromosome positions where selection candidates are identified by four methods. Black triangles show the approximate locations of centromeres. The Y-axis is the chromosome position (cM); the X-axis is the number of selection loci identified.

The selection map revealed that selection candidates were clustered (less than 1 cM) in the wheat genome (Fig. 7). Of the 7,984 selection candidate loci, 5,317 (66.6%) were distributed in 515 clusters, 10.3 loci per cluster on average, suggesting that these clusters were active or ‘hot’ selection regions. These regions have made significant contributions to wheat domestication and improvement. Therefore, they will continue to be important regions for wheat evolutionary studies and breeding. For example, 109 SNPs were identified as selection candidates at position 61 on chromosome 1B with which the traits Ph, TGW and Y were significantly associated (Figs 6 and 7). The clusters were widely distributed in the wheat genome, but not evenly. More clusters (211) were present in the B sub-genome with less (115) in the D sub-genome. The largest number (43) of clusters was in chromosome 5A and least (8) in chromosome 4D (Table 4); the numbers of loci per cluster was highest for chromosomes 1B (635) and 5B (623) and least for 4D (41). There were six clusters, including three in 1B, and one each in 2B, 5B and 6A where more than one hundred selection loci were included in each cluster. More clusters were located around the centromeric regions, suggesting that these regions were also significant targets of selection although gene densities are much lower in those regions^32^.

## Discussion

In this study 34,317 SNP loci were screened by four methods for evidence of selection during evolution; 7,984 non-redundant loci were detected as selection candidates, accounting for 23.3% of all tested loci. Across different statistical analyses the selected loci showed differences in patterns of genetic differentiation or diversity variation among the three populations.

### More loci were selected in crop evolution

The proportions and identities of selected loci in crop evolution are very important issues. In previous studies, the percentages of genome-wide selection signatures ranged from 5% in maize^16^ to 7% in sunflower^17^. Moreover, only loci with reduced diversity were considered in those reports resulting in lower ratios of selection outliers. Whole-genome analysis in rice revealed that more than 10% genes were positively selected during differentiation of cultivars 93-11 and Nipponbare^33^. This indicated that loci with reduced diversity or those identified by only one method might be insufficiently representative of the total numbers of selection candidates.

Obviously, selection caused losses in diversity as reflected by reduced allele numbers through elimination of unfavorable alleles. Well-known selected genes, for example *Q* in wheat^34^, *tb1*^35^ and *tga1*^36^ in maize, and *sh4* in rice^37^, have reduced diversity in derived populations. In the present study, loss of genetic diversity was observed for 1,641 loci (Supplementary Dataset 2), or 20.6% of all identified selection candidates. The diversity changes at these loci were consistent with models presented previously^4^,^14^.

Selection can also increase diversity in derived populations due to increased allele numbers and/or balanced allelic frequencies. Increasing allele numbers in crop plants might be required for adaptation to new environments. All crops originate from localised areas^4^, and to adapt to a wider range of environments additional alleles related to environmental adaptation must be generated. The wild diploid ancestors of wheat occur in parts of the Fertile Crescent; for *T. urartu* the current location is the Karacadağ mountain region of southeastern Turkey; for *Ae. tauschii* it is to the south and west of the Caspian Sea; wild emmer (*T. dicoccoides*) currently occurs in the Jordan Valley in Israel and neighbouring regions^4^,^38^. Modern wheat varieties are now planted worldwide, thus the range in diversity of adaptation-related or ecotype-related genes in wild populations is likely to be comparatively small. When wheat spread throughout the world, novel adaptation-related alleles at loci such as those for photoperiod, vernalization and stress tolerance were selected in the adaptation process, resulting in previously rare alleles becoming frequent in modern varieties. Our data support this speculation and show 1,659 loci with increased diversity at candidate loci, such as domestication loci in the *Vrn-1* and *Ppd-B1* regions (Fig. 2).

Genetic diversity of some selected loci could remain statistically non-variable in derived populations compared with ancestral populations. However, the frequencies of certain alleles were quite different between populations, presumably resulting from balanced allele distributions. In this study, 4,640 selection loci did not show significant differences in diversity between populations, but the predominating alleles and corresponding frequencies in different populations were quite different (Supplementary Dataset 1). This was characterized by some improvement loci. In this study, locus wsnp_Ex_c55777_58153636 also in the *Vrn-A1* region was identified as an improvement signature by the *F*_ST_ and *Freq* methods. The frequency of allele A at this locus increased from 1.0% in population *W* to 22.5% in *L* and to 84.5% in *M*, indicating high selection pressure on allele A during evolution. However, the diversity at this locus between groups did not differ significantly. The same result was obtained for *Vrn-B1* (wsnp_Ex_c29304_38355434 ~ wsnp_Ku_c21770_31551190).

Out results were consistent with that reported in maize^8^ where divergent regions with statistically significant reductions and increases in expected heterogeneity were about 7 and 35.7%, respectively, and the remaining 57.1% of regions did not show significant changes in levels of expected heterogeneity. These results indicated that diversity at loci under selection was not only lost but also increased or remained constant.

We also recognize that there are false positive selection loci detected by any approach, increasing the proportion of selection loci. Generally, the more methods used to detect selection loci, the more reliable the candidates will be. Therefore, more efficient approaches are needed. In fact, the loci detected by any approach are only candidates and further analysis is needed to prove their function.

### Importance of a selection map of wheat domestication and improvement

The selection map clearly demonstrated the distribution of selection candidates during wheat evolution, including selection hotspots and cold spots (Figs 6 and 7). Such maps show genetic regions under selection pressure during wheat domestication and improvement and selection that occurred during both or in the separate phases. Domestication and improvement candidates tended to occur in clusters and were independent from each other across the genome, implying that the selection targets of the two processes were different. Domestication put more selection pressure than improvement on the D sub-genome, presumably due to the population bottleneck following polyploidization. In addition, the map clearly shows that centromeric regions were also selection targets, and that some chromosome regions were untouched by selection. Because only landraces and modern varieties were analysed by GWAS, much attention should be given to regions under selection only during domestication. These regions should be potentially important in traits for adaptation to new environments or for agronomic traits. The selection map was not only valuable for reviewing selection history, but more importantly, it suggests targets for future novel gene selection or allele discovery, genomic selection and wheat design.

### Improving D sub-genome diversity by using synthetic wheat in wheat improvement

Progress in yield improvement by wheat breeding following the Green Revolution has been slow, and fails to meet projected needs for a growing world population and environmental changes. Increased yields are a first priority for wheat breeders. Common wheat is hexaploid and is comprised of the homoeologous A, B and D sub-genomes with similar gene numbers, or possibly even more genes, in the D sub-genome than in the A sub-genome^39^,^40^. However, less selection loci were detected in the D sub-genome in the present study. Results from this study and previous reports^41^,^42^ indicated that the D sub-genome made less contributions in modern breeding, implying that the D sub-genome has a high potential for future wheat improvement. This lower contribution is caused by low diversity in the D sub-genome in common wheat resulting from the bottleneck caused when hexaploid wheat arose from a single (or extremely few) spontaneous hybridizations of tetraploid wheat with *Ae. tauschii* 8,000–10,000 years ago^43^,^44^. However, *Ae. tauschii* is rich in genetic diversity^38^,^45^. Therefore, mining agronomically important genes from the D sub-genome will be important in future wheat improvement^46^. The use of synthetic wheat and synthetic wheat introgression lines (IL) should be an efficient approach to expand the diversity of the D sub-genome in hexaploid wheat. In the present study, we detected 17 loci affecting yield in the A and B sub-genomes, and 14 loci in a D sub-genome derived IL population, although the number of original SNP markers in the D sub-genome was much less than those from the A and B sub-genomes. Among the 14 D sub-genome loci 3 alleles originating from *Ae. tauschii* increased yield in a commercial wheat variety background by more than 10%. Chinese wheat variety Chuanmai 42 was bred by using a synthetic wheat as one parent, and its yield was 22.7% higher than the check cultivar Chuanmai 107^47^. UK scientists also reported a ‘super’ wheat line with 30% higher yield potential than the check (http://www.bbc.com/news/uk-22498274). These encouraging reports suggest that *Ae. tauschii* should be more widely exploited in future wheat improvement. As suggested recently^48^, further development of synthetic wheats and use of ILs may provide the needed breakthroughs in wheat breeding.

## Methods

### Plant materials, DNA extraction and genotyping

The wheat samples used for genome-wide scanning for selection signatures comprised three groups, viz. 188 wild relatives (*W*), 84 landrace cultivars (*L*) and 429 modern cultivars (*M*) (Supplementary Dataset 7). The wild lines included 96 wild emmer (*Triticum dicoccoides*) and 92 goatgrass (*Aegilops tauschii*) accessions. But when we do experiment, we combined DNA of one wild emmer wheat line with one goat grass line into one well, which made 96 combinations with four goatgrass repeated. Therefore, we described 96 wild relative combinations as wild group. Landraces and modern varieties used here are or were major cultivars in China, and were grown in crop season 2011–2012 at four locations, viz., Beijing (E1, 40°N, 116°E), and Jiaozuo (E2, 35°N, 113°E), Luoyang (E3, 33°N, 111°E) and Xinxiang (E4, 35°N, 113°E) in Henan province as single 0.5 × 2 m plots. These cultivars were also grown in crop seasons 2012–2013 (E5) and 2013–2014 (E6) in Xinxiang as single 1.5 × 3 m plots. The phenotypic traits investigated included plant height (Ph), spike length (SL), spikelet number per plant (SLN), grain number per spike (GN), thousand-grain weight (TGW) and tiller number per plant (T), heading time (Ht), maturity time (Mt) and powdery mildew (PM) response. Each phenotypic trait value represented the mean of at least six plants per line. In addition the yield per hectare (Y) was measured for each wheat cultivar during the 2013–2014 and 2014–2015 seasons.

DNA was isolated from the leaves of two-week-old seedlings using a DNA extraction kit (CN. DP321, Tiangen Biotech Co., Ltd.). These DNA samples were genotyped by the Illumina wheat 90K iSelect Assay^26^. SNP clustering and genotype calling were performed using GenomeStudio v2011.1 software (Illumina). As previously described^25^,^28^ genotyping of polyploid wheat using the 90K SNP chip was complicated by the presence of homoeologous and paralogous gene copies among assays. Therefore, one SNP in the 90K chip might not be typically bi-allelic, and manual adjustments of the clustering patterns were necessary to ensure accurate genotyping.

### Genetic diversity and measures of differentiation

SNP allele frequency and unbiased genetic diversity (*He*) were estimated for each locus using Powermarker v3.25^49^. Pairwise population *F*_ST_-values were calculated in Arlequin 3.5^21^ and tested against a null distribution obtained by 100,000 permutations of genotypes between wild and landrace (*WL*) and between landrace and modern (*LM*) cultivar accessions. Tajima’s D was estimated by using TASSEL v5.0^50^ with a sliding window size of five SNPs and step size of one SNP along all 21 chromosomes.

#### Population structure

To illustrate the relationships between wild, landrace and modern variety accessions, a UPGMA tree was constructed based on a Sokal-Michener matching dissimilarity matrix using the program DARwin v5.0.155^51^. Branch support was determined by bootstrapping (1,000 replicates). In addition, possible population structure between landrace and modern varieties was also determined using the program STRUCTURE ver2.2^52^ with the admixture and correlated allele frequencies model. Program run length was 10,000 with ten iterations to test for *K* values in the range of 1–3. The likely number of subpopulations present was estimated following Evanno *et al*.^53^.

### Pairwise population tests to detect loci under selection

To identify linked candidate loci in genomic regions that had undergone selection during wheat domestication (*WL*) and improvement (*LM*), four methods were applied to identify outliers.

The first method detected outliers under selection from genetic structure analysis as described in Arlequin 3.5^21^ where a hierarchical island model is used to perform coalescent simulations leading to the joint null distribution of hierarchical F-statistics and heterogeneities, from which locus-specific p-values are estimated. The hierarchical population structure is defined in the structure section. For detection of outliers under selection during wheat domestication and improvement *WL* and *LM* pairs were set and 20,000 coalescent simulations were performed to obtain null distributions of F-statistics. One hundred groups and 10 demes per group were simulated and a 5% quantile criterion was used. Loci with unusual *F*_ST_ values conditional on heterozygosity/heterogeneity were regarded as potentially under selection.

The second approach, called the *lnRH-test*, was based on genetic variation calculated by the ratio of genetic diversity in two populations under comparison^19^. *lnRH* was calculated as suggested by Schlotterer and Dieringer^54^:


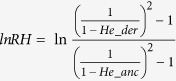
, where *He_der* and *He_anc* correspond to variances in genetic diversity of the derived and ancestral populations being compared, as described by Chapman *et al*.^17^. In cases where monomorphism occurred in the ancestral population, one allele in the monomorphic ancestral population was changed to a different allele as suggested^54^. The *lnRH* values were normalized and standardized and differences between the expected distribution and empirical confidence intervals at p = 0.05 were determined. The *lnRH* method has been used in identifying selection signatures based on SNP markers^29^.

The third method was based on frequency comparisons between groups. It was assumed that selection acts on alleles of each locus and changes allele frequency. In this study, we applied a simple statistical method to test the significance of differences in variances between populations. The null hypothesis was no difference in frequency distribution between two populations. The *μ* value was calculated according to Gai^55^:


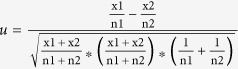
, where x1 and x2 were numbers of alleles for each locus, and n1 and n2 were the numbers of loci compared between populations. SNPs with absolute *μ* values larger than 11 between populations were considered to be selection outliers. Extremely higher *μ* values were used here to eliminate the number of false positives as much as possible.

The fourth method was based on genome-wide association analysis (GWAS). This method was suggested as a bottom-up approached to identify selection signatures^22^. In this study trait data were collected for cultivars, and only landrace and modern varieties were analyzed. A total of 22,533 SNPs with known chromosome positions were used. We expected to find loci that significantly affected phenotypic differences between populations *L* and *M*. GWAS was calculated by the Anderson-Darling test^56^. A Q-Matrix was generated from STRUCTURE using 3,943 SNPs uniquely distributed on all 21 chromosomes. Bonferroni-corrected thresholds of α = 0.05 as cut-off values were used; at α = 0.05, the Bofferoni-corrected threshold for the p values were 2E-06 with a corresponding −logp value of 5.654. An additional GWAS sample set that included 115 introgression lines was also used to identify trait-marker associations. Synthetic wheat accession Am3 as donor and Chinese cultivar Laizhou 953 as recipient were crossed and backcrossed to build the introgression lines^57^. Because Am3 was synthesized by crossing *T. carthlicum* with *Ae. tauschii*, association results based on introgression lines were complementary to the natural population composed of landraces and modern varieties.

## Additional Information

**How to cite this article:** Gao, L. *et al*. Candidate loci involved in domestication and improvement detected by a published 90K wheat SNP array. *Sci. Rep.* **7**, 44530; doi: 10.1038/srep44530 (2017).

**Publisher's note:** Springer Nature remains neutral with regard to jurisdictional claims in published maps and institutional affiliations.

## Supplementary Material

Supplementary Information

Supplementary Dataset 1

Supplementary Dataset 2

Supplementary Dataset 3

Supplementary Dataset 4

Supplementary Dataset 5

Supplementary Dataset 6

Supplementary Dataset 7

## Figures and Tables

**Table 1:** Summary of selection candidates identified by different methods during wheat domestication and improvement.

		Domestication			Total	Improvement				Total^1^	No. loci analyzed
		*F*_ST_	*lnRH*	*Freq*		*F*_ST_	*lnRH*	*Freq*	GWAS		
	1A	141	29	92	150	38	16	42	138	58	1293
	2A	150	98	31	174	167	14	161	67	185	1197
	3A	129	17	101	137	81	18	78	172	100	1036
	4A	96	38	71	121	14	41	31	31	60	886
	5A	125	56	89	168	41	53	42	161	91	1328
	6A	146	138	38	199	1	15	2	285	17	1198
	7A	110	31	99	140	92	36	94	53	131	1370
	**A sub-genome**	**897**	**407**	**521**	**1089**	**434**	**193**	**450**	**907**	**642**	**8308**
	1B	163	55	84	177	18	356	19	460	377	2089
	2B	155	56	60	168	49	42	59	318	98	1779
	3B	91	20	52	96	18	64	22	79	86	1160
	4B	155	37	146	196	44	105	45	22	152	948
	5B	177	21	157	209	53	178	49	459	233	1691
	6B	89	46	33	108	79	25	68	64	102	1451
	7B	84	40	53	108	70	45	70	79	102	1307
	**B sub-genome**	**914**	**275**	**585**	**1062**	**331**	**815**	**332**	**1481**	**1150**	**10425**
	1D	58	28	36	75	0	15	0	20	15	575
	2D	77	42	50	106	4	163	4	15	167	863
	3D	62	17	48	78	8	16	5	17	24	393
	4D	13	14	21	35	1	21	1	5	22	173
	5D	39	91	55	147	0	9	0	8	9	680
	6D	27	23	17	50	3	6	2	45	9	306
	7D	38	128	51	182	7	5	7	9	12	810
	**D sub-genome**	**314**	**343**	**278**	**673**	**23**	**235**	**19**	**119**	**258**	**3800**
Total	Known positions	2125	1025	1384	2824	788	1243	801	2507	2050	22533
	Unknown positions	663	655	355	1187	209	465	234		647	11784
	**All**	**2788**	**1680**	**1739**	**4011**	**997**	**1708**	**1035**	**2507**	**2697**	**34317**

^1^The number of selection candidates identified by *F*_ST_, *lnRH* and *Freq*.

**Table 2:** Comparison of agronomic traits^1^ between groups *L* and *M*.

Parameter^2^	T	Ph	SL	SLN	GN^3^	TGW	Ht	Mt	PM	Y
Mean	10/8	128/86.7	9.1/8.9	19/18	47/45	31.9/43.4	191.3/187.5	230.3/228	3/2.6	1.9/2.9
Vc (%)	34/33	10.5/18.8	24.1/18.6	10.7/10.3	20/19.1	20.4/15.9	3.2/3.4	0/1.9	36.2/46.1	25.8/19.3
Min	3.7/3	72.4/53.8	4.8/4.3	12/13	22/21	15.7/18.8	167/167	217/197	0/0	0/0.4
Max	20/19	162.7/145.8	18.3/25.5	29/29	75.2/89.3	52.5/68.9	211/210	245/244	4/4	3/4
V_M/L_(%)	−20	−32	−2	−5	−2	36	−2	−1	13	53
T-test(p value)	0	0	0.012	0	0	0	0	0	0	0

^1^T, tiller number; Ph, plant height; SL, spike length; SLN, spikelet number; GN, grain number; TGW, thousand grain weight; Ht, heading time; Mt, mature time; PM, powdery mildew resistance; Y, yield.

^2^Numbers left and right of “/” were the traits of landraces and of modern varieties, respectively.

^3^Trait GN was significantly different between *L* and *M* in three environments. V_M/L_ (%) is the trait variations of population *M* compared with that of *L*.

**Table 3:** Summary of selection loci identified by different methods.

	Total	Common	Unique	*lnRH*	*Freq*	GWAS
*F*_ST_	3568	3049	519	801	2361	419
*lnRH*	3344	1306	2038	—	229	559
*Freq*	2674	2435	239	—	—	304
GWAS	2507	893	1614	—	—	—

**Table 4:** Numbers of selection clusters and their distribution in the wheat genome.

Homoelogeous group	A			B			D		
	Loci	Clusters	Average	Loci	Clusters	Average	Loci	Clusters	Average
1	249	36	6.9	635	37	17.2	73	15	4.9
2	308	19	16.2	465	39	11.9	236	28	8.4
3	296	28	10.6	186	29	6.4	63	13	4.8
4	156	18	8.7	289	22	13.1	41	8	5.1
5	314	43	7.3	623	39	16.0	124	19	6.5
6	411	24	17.1	207	21	9.9	67	11	6.1
7	239	21	11.4	199	24	8.3	136	21	6.5
	1973	189	10.4	2604	211	12.3	740	115	6.4
