# Supplementary material for: Candidate loci involved in domestication and improvement detected by a published 90K wheat SNP array
Source: Sci Rep. 2017 Mar 22;7:44530. doi: 10.1038/srep44530 (PMC5361097; doi:10.1038/srep44530)
Supplement: Supplementary Information [file srep44530-s1.pdf]

Candidate loci involved in domestication and improvement detected by a published 90k wheat SNP array

Lifeng Gao[1], Guangyao Zhao[1], Dawei Huang[1], and Jizeng Jia[1*]

[1]Key Laboratory of Crop Gene Resources and Germplasm Enhancement, MOA, the National Key Facility for Crop Gene Resources and Genetic Improvement, Institute of Crop Science, CAAS, Beijing, China, 100081

*Correspondence and requests for materials should be addressed to J.Z.J (email: jiajizeng@caas.cn)

Email: jiajizeng@caas.cn

Supporting information

Supplementary Figure 1. Population structure of three populations in the K=3 model. Each accession was depicted by a vertical line segmented into 3 colored sections: blue, red and green for population W, L and M respectively. The length of each section was proportional to the estimated membership coefficient (Q) of the accession to each cluster.

Supplementary Figure 2. GWAS of the phenotype of tiller number (T) by Anderson–Darling test. Red and blue line indicated the Bonferroni corrected threshold of (-log10(P)=6.35, a = 0.01) and (-log10(P)=5.65, a = 0.05) respectively.

Supplementary Figure 3. GWAS of the phenotype of spike length (SL) by Anderson–Darling test. Red and blue line indicated the Bonferroni corrected threshold of (-log10(P)=6.35, a = 0.01) and (-log10(P)=5.65, a = 0.05) respectively.

Supplementary Figure 4. GWAS of the phenotype of spikelet number per spike (SLN) by Anderson–Darling test. Red and blue line indicated the Bonferroni corrected threshold of (-log10(P)=6.35, a = 0.01) and (-log10(P)=5.65, a = 0.05) respectively.

Supplementary Figure 5. GWAS of the phenotype of grain number (GN) by Anderson–Darling test. Red and blue line indicated the Bonferroni corrected threshold of (-log10(P)=6.35, a = 0.01) and (-log10(P)=5.65, a = 0.05) respectively.

Supplementary Figure 6. GWAS of the phenotype of thousand grain weight (TGW) by Anderson–Darling test. Red and blue line indicated the Bonferroni corrected threshold of (-log10(P)=6.35, a = 0.01) and (-log10(P)=5.65, a = 0.05) respectively.

Supplementary Figure 7. GWAS of the phenotype of yield (Y) by Anderson–Darling test. Red and blue line indicated the Bonferroni corrected threshold of (-log10(P)=6.35, a = 0.01) and (-log10(P)=5.65, a = 0.05) respectively.

Supplementary Figure 8. GWAS of the phenotype of plant height (Ph) by Anderson–Darling test. Red and blue line indicated the Bonferroni corrected threshold of (-log10(P)=6.35, a = 0.01) and (-log10(P)=5.65, a = 0.05) respectively.

Supplementary Figure 9. GWAS of the phenotype of heading time (Ht) by Anderson–Darling test. Red and blue line indicated the Bonferroni corrected threshold of (-log10(P)=6.35, a = 0.01) and (-log10(P)=5.65, a = 0.05) respectively.

Supplementary Figure 10. GWAS of the phenotype of mature time (Mt) by Anderson–Darling test. Red and blue line indicated the Bonferroni corrected threshold of (-log10(P)=6.35, a = 0.01) and (-log10(P)=5.65, a = 0.05) respectively.

Supplementary Figure 11. GWAS of the phenotype of resistance to powdery mildew (PM) by Anderson–Darling test. Red and blue line indicated the Bonferroni corrected threshold of (-log10(P)=6.35, a = 0.01) and (-log10(P)=5.65, a = 0.05) respectively.

Supplementary Figure 12. Selection map of wheat during domestication and improvement. Red, green and purple lines denote candidates subjected to selection during domestication, improvement and both processes, respectively. Centromeric regions are indicated by black dots in accordance with Cavanagh et al.[25] Detailed information on the mapped loci is provided in Supplementary dataset 1 to 4.

Supplementary Figures

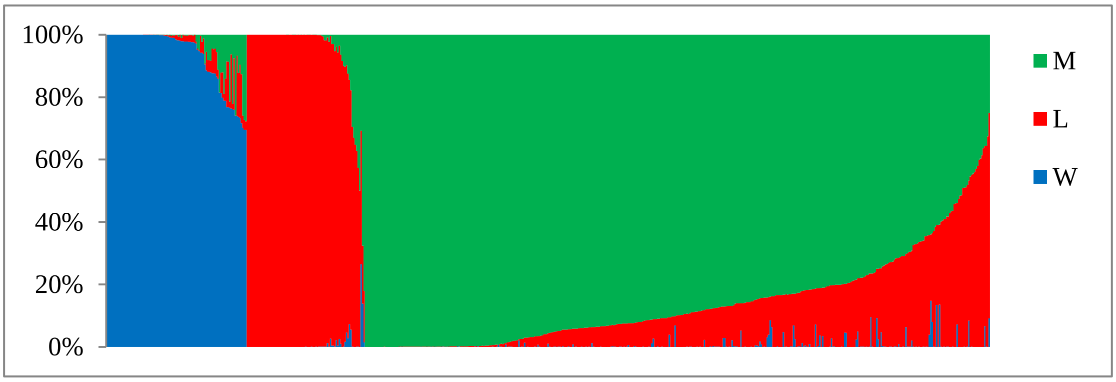

Supplementary Figure 1. Population structure of three populations in the K=3 model. Each accession was depicted by a vertical line segmented into 3 colored sections: blue, red and green for population *W*, *L* and *M* respectively. The length of each section was proportional to the estimated membership coefficient (Q) of the accession to each cluster.

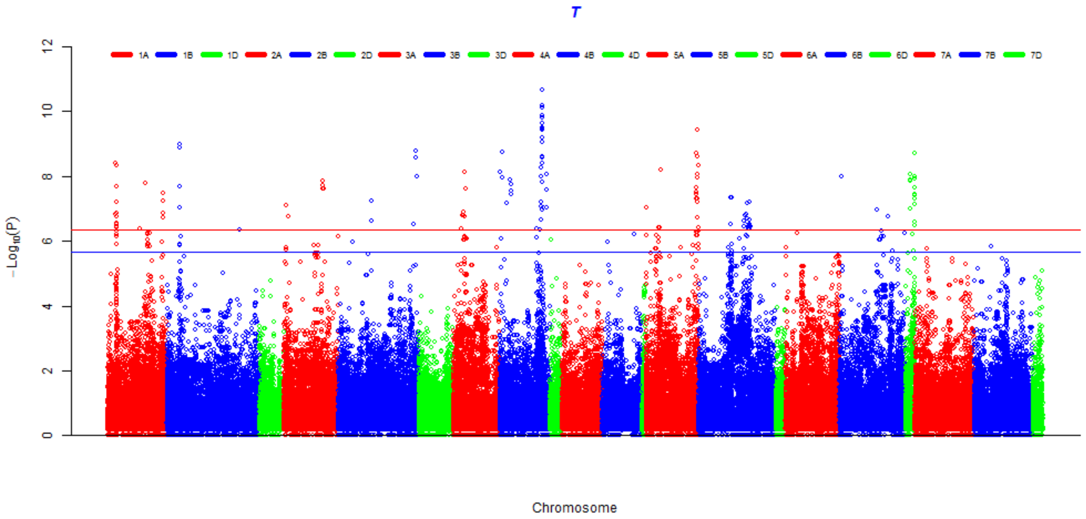

Supplementary Figure 2. GWAS of the phenotype of tiller number (T) by Anderson–Darling test. Red and blue line indicated the Bonferroni corrected threshold of (-log10(P)=6.35, a = 0.01) and (-log10(P)=5.65, a = 0.05) respectively.

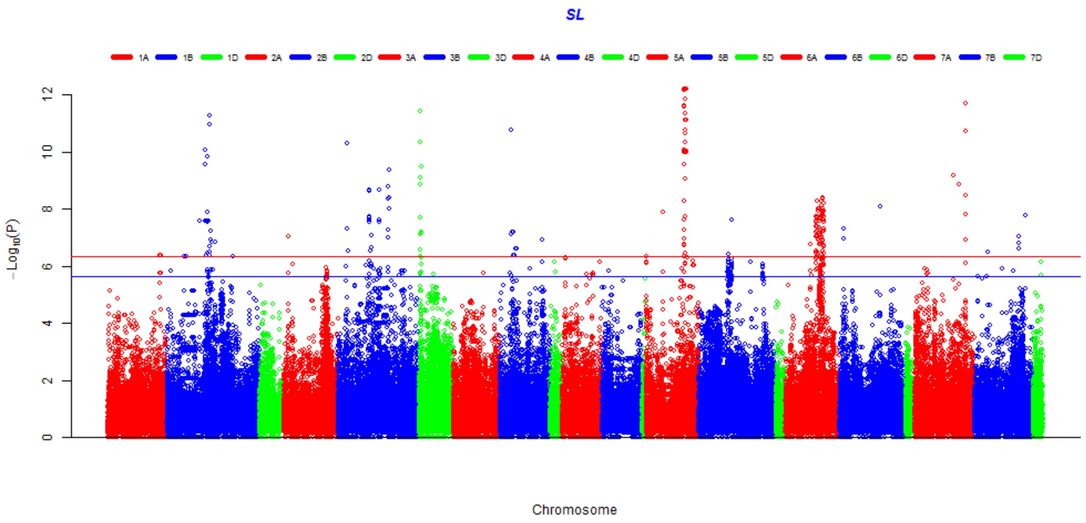

Supplementary Figure 3. GWAS of the phenotype of spike length (SL) by Anderson–Darling test. Red and blue line indicated the Bonferroni corrected threshold of (-log10(P)=6.35, a = 0.01) and (-log10(P)=5.65, a = 0.05) respectively.

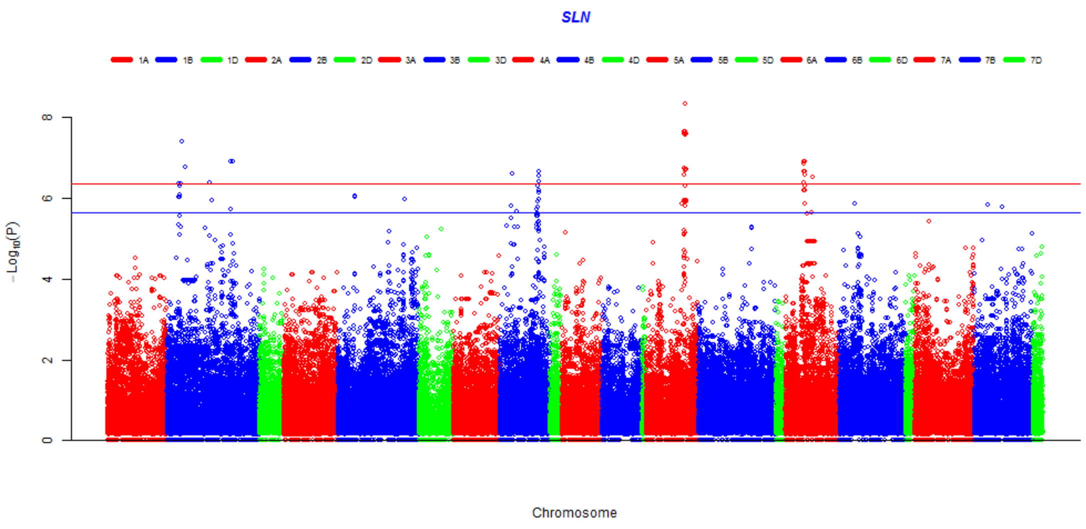

Supplementary Figure 4. GWAS of the phenotype of spikelet number per spike (SLN) by Anderson–Darling test. Red and blue line indicated the Bonferroni corrected threshold of (-log10(P)=6.35, a = 0.01) and (-log10(P)=5.65, a = 0.05) respectively.

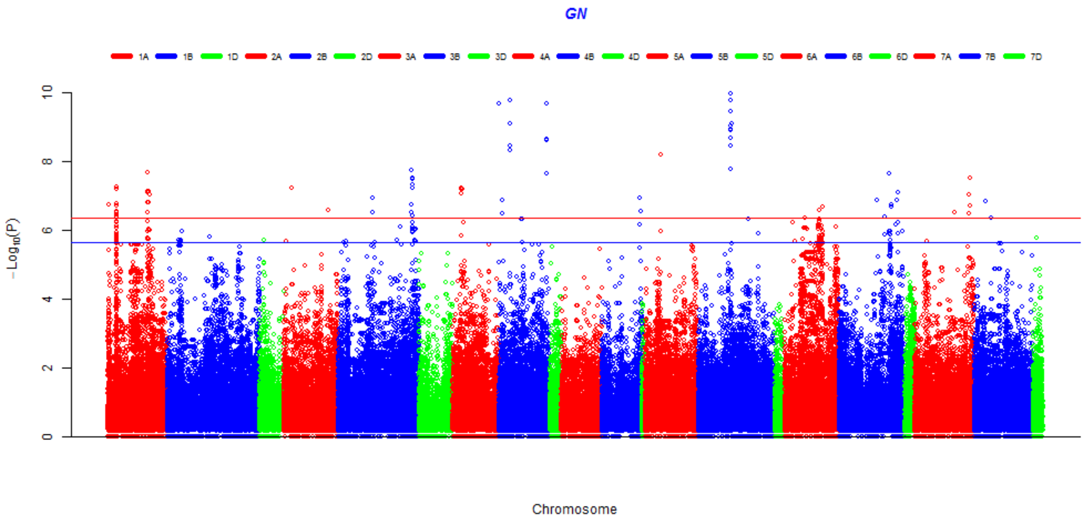

Supplementary Figure 5. GWAS of the phenotype of grain number (GN) by Anderson–Darling test. Red and blue line indicated the Bonferroni corrected threshold of (-log10(P)=6.35, a = 0.01) and (-log10(P)=5.65, a = 0.05) respectively.

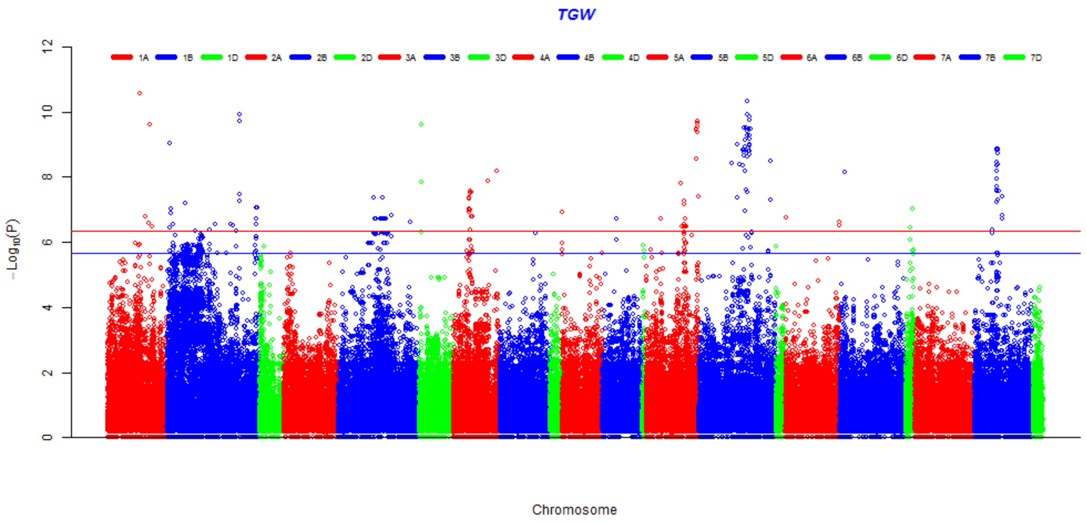

Supplementary Figure 6. GWAS of the phenotype of thousand grain weight (TGW) by Anderson–Darling test. Red and blue line indicated the Bonferroni corrected threshold of (-log10(P)=6.35, a = 0.01) and (-log10(P)=5.65, a = 0.05) respectively.

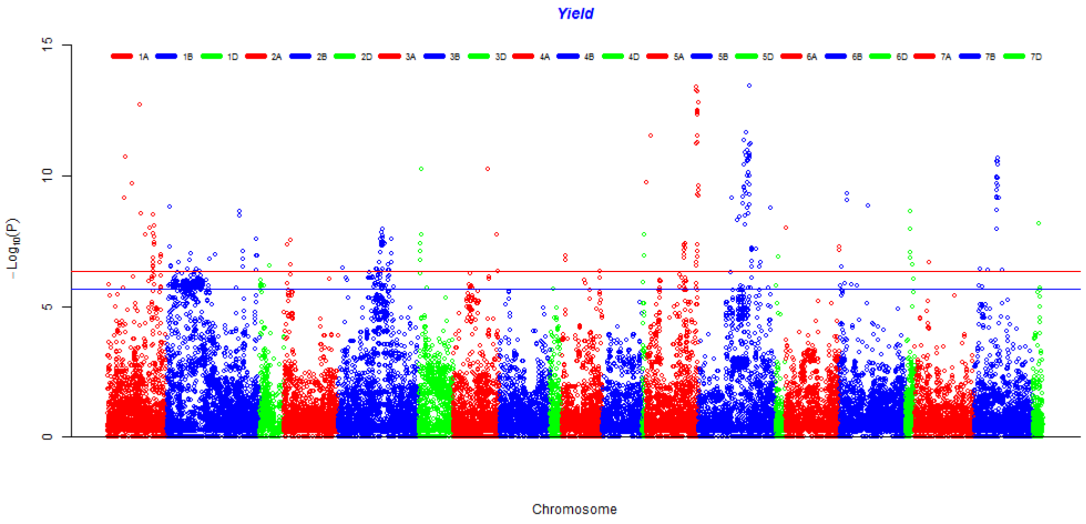

Supplementary Figure 7. GWAS of the phenotype of yield (Y) by Anderson–Darling test. Red and blue line indicated the Bonferroni corrected threshold of (-log10(P)=6.35, a = 0.01) and (-log10(P)=5.65, a = 0.05) respectively.

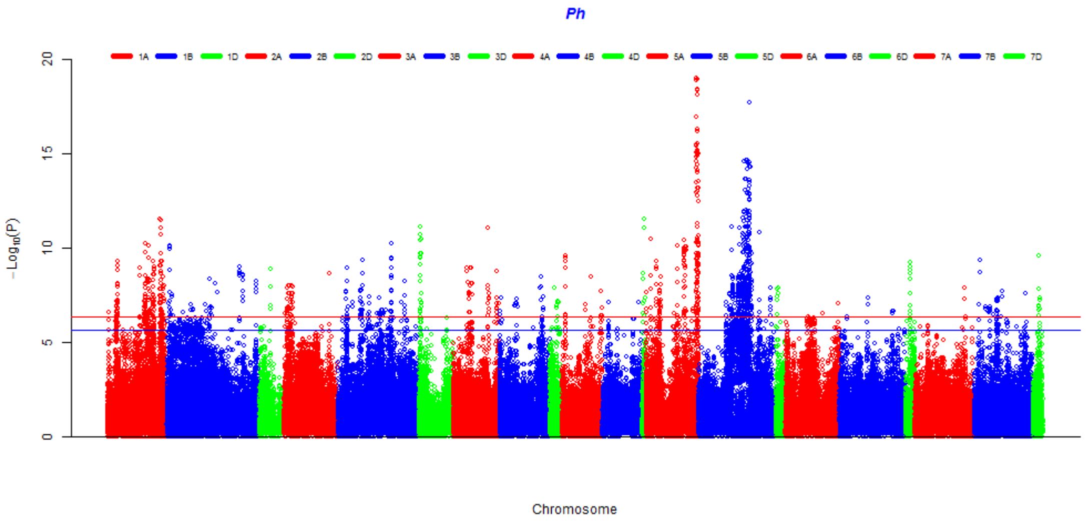

Supplementary Figure 8. GWAS of the phenotype of plant height (Ph) by Anderson–Darling test. Red and blue line indicated the Bonferroni corrected threshold of (-log10(P)=6.35, a = 0.01) and (-log10(P)=5.65, a = 0.05) respectively.

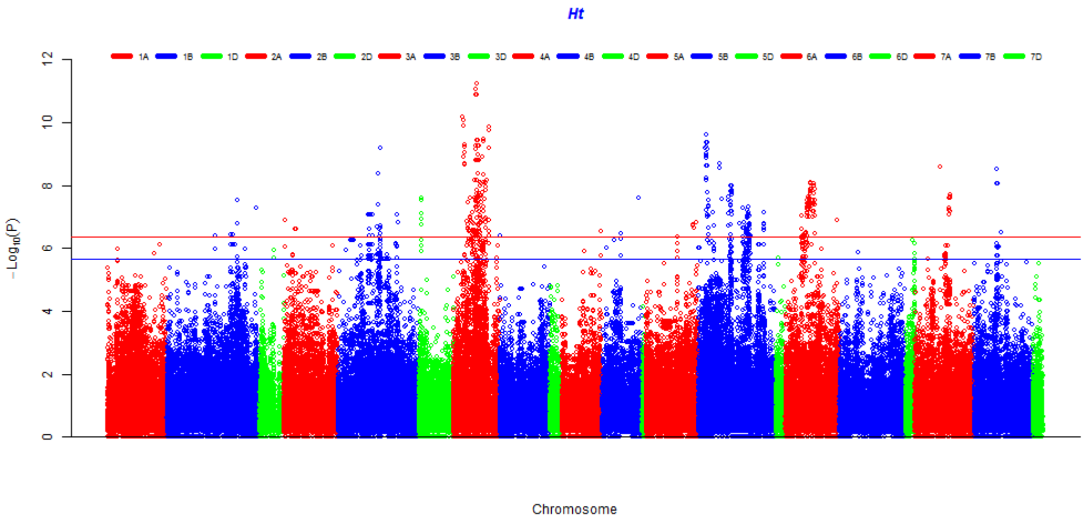

Supplementary Figure 9. GWAS of the phenotype of heading time (Ht) by Anderson–Darling test. Red and blue line indicated the Bonferroni corrected threshold of (-log10(P)=6.35, a = 0.01) and (-log10(P)=5.65, a = 0.05) respectively.

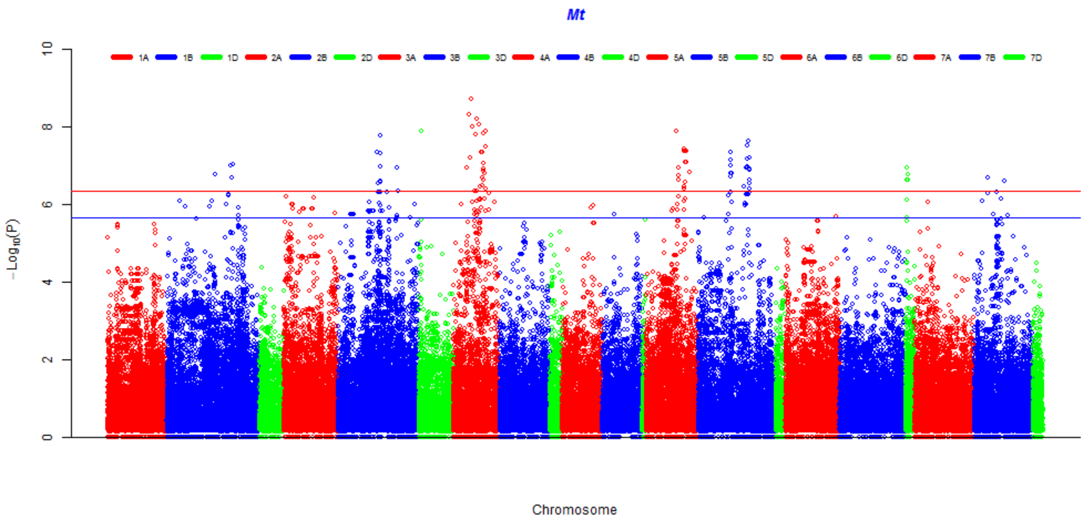

Supplementary Figure 10. GWAS of the phenotype of mature time (Mt) by Anderson–Darling test. Red and blue line indicated the Bonferroni corrected threshold of (-log10(P)=6.35, a = 0.01) and (-log10(P)=5.65, a = 0.05) respectively.

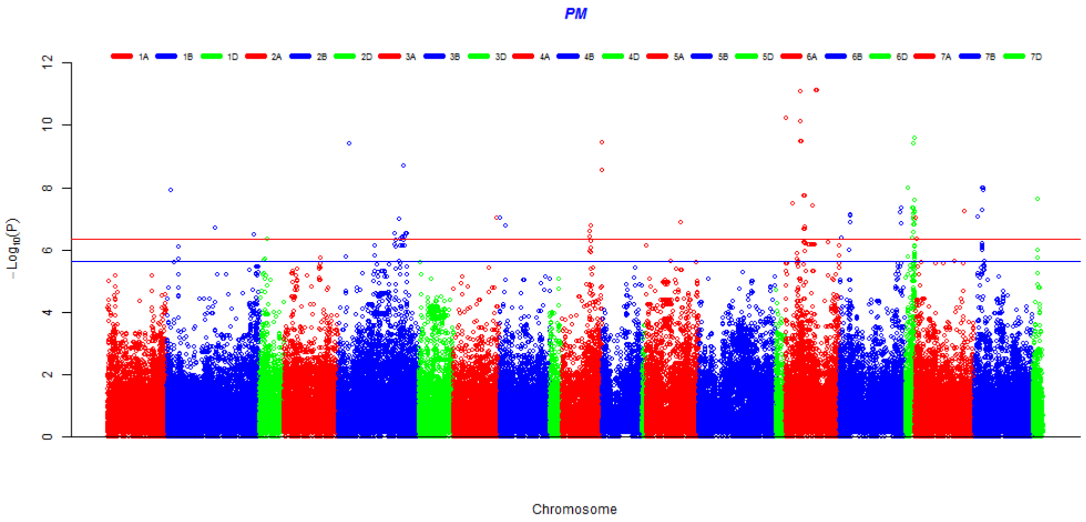

Supplementary Figure 11. GWAS of the phenotype of resistance to powdery mildew (PM) by Anderson–Darling test. Red and blue line indicated the Bonferroni corrected threshold of (-log10(P)=6.35, a = 0.01) and (-log10(P)=5.65, a = 0.05) respectively.

Supplementary Figure 12. Selection map of wheat during domestication and improvement. Red, green and purple lines denote candidates subjected to selection during domestication, improvement and both processes, respectively. Centromeric regions are indicated by black dots in accordance with Cavanagh et al.[25] Detailed information on the mapped loci is provided in Supplementary Datasets 1 to 4.
